# Development and validation of a risk model for noninvasive detection of cancer in oral potentially malignant disorders using DNA image cytometry

**DOI:** 10.20892/j.issn.2095-3941.2020.0531

**Published:** 2021-08-15

**Authors:** Chenxi Li, Yongmei Zhou, Yiwen Deng, Xuemin Shen, Linjun Shi, Wei Liu

**Affiliations:** 1 Department of Oral Mucosal Diseases, Shanghai Ninth People’s Hospital, Shanghai Jiao Tong University School of Medicine, College of Stomatology, Shanghai Jiao Tong University, National Center for Stomatology, National Clinical Research Center for Oral Diseases, Shanghai Key Laboratory of Stomatology, Shanghai 200011, China; 2 Department of Oral and Maxillofacial-Head and Neck Oncology, Shanghai Ninth People’s Hospital, Shanghai Jiao Tong University School of Medicine, Shanghai 200011, China

**Keywords:** Oral squamous cell carcinoma, potentially malignant disorders, DNA aneuploidy, image cytometry, dysplasia

## Abstract

**Objective:**

To elucidate whether DNA aneuploidy was an independent discriminator for carcinoma within oral potentially malignant disorders (OPMDs), and further establish and validate a risk model based on DNA aneuploidy for the detection of oral cancer.

**Methods:**

A total of 810 consecutive patients with OPMD were prospectively enrolled from March 2013 to December 2018, and divided into a training set (*n* = 608) and a test set (*n* = 202). Brushing and biopsy samples from each patient were processed by DNA- DNA image cytometry and histopathological examination, respectively.

**Results:**

DNA aneuploidy of an outside DNA index ≥ 3.5 in OPMD was an independent marker strongly associated with malignant risk [adjusted odds ratio: 13.04; 95% confidence interval (CI): 5.46–31.14]. In the training and test sets, the area under the curve (AUC) was 0.87 (95% CI: 0.82–0.91) and 0.77 (95% CI: 0.57–0.97), respectively, for detecting carcinoma in OPMD patients. The independent risk factors of lateral/ventral tongue and non-homogenous type combined with a risk model built with a multivariate logistic regression revealed a more favorable diagnostic efficacy associated with the training set (AUC: 0.93; 95% CI: 0.91–0.96) and test set (AUC: 0.94; 95% CI: 0.90–0.98). The sensitivity and specificity of carcinoma detection within OPMD was improved to 100% and 88.1%, respectively.

**Conclusions:**

This large-scale diagnostic study established a risk model based on DNA aneuploidy that consisted of a noninvasive strategy with lateral/ventral tongue and non-homogenous features. The results showed favorable diagnostic efficacy for detecting carcinoma within OPMD, irrespective of the clinical and pathological diagnoses of OPMD. Multicenter validation and longitudinal studies are warranted to evaluate community practices and clinical applications.

## Introduction

Oral squamous cell carcinoma (OSCC) accounts for over 90% of oral cancer, which represents the most common cancers and a leading cause of cancer-related death worldwide^1,2^. Previous reports indicate that 19.5%–48.0% of patients with OSCC are associated with concomitant leukoplakia as the main subtype of oral potentially malignant disorders (OPMDs)^3–5^. Although scalpel biopsy and histopathological examination remain the gold standard for detecting malignant changes in OPMD patients, histological assessment remains insufficient and highly subjective in clinical practice^6,7^. Therefore, additional objective diagnostic techniques are required for earlier detection of carcinoma in OPMD patients and to contribute to the surveillance of OPMD progression^8^.

A loss of heterozygosity and expression of cancer stem cell markers have been substantially validated to be of prognostic value for high risk OPMD^9,10^. Biomarker applications represent a biopsy-based strategy that incorporates possible sampling errors in the detection of malignant changes^6,7^. Moreover, it remains uncertain whether an incisional biopsy sample from a suspicious lesion is reliable and representative of the histological findings of the whole lesion^6^. Moreover, invasive sequential biopsies have limited reproducibility for the surveillance of patients with oral suspicious lesions. Therefore, the development of novel methods of detection for OPMD using models based on objectively-assessed genetic and molecular alterations are required to facilitate the diagnoses and treatments of at-risk OPMD and early OSCC^11^.

Aneuploidy is a cancer-type-specific oncogenic event that may have clinical relevance as a prognostic marker and potential therapeutic target^12^. The DNA ploidy status determined by image cytometry (ICM) is an objective additional diagnostic technique that can be used to automatically measure nuclear DNA content^13^. Although DNA aneuploidy is known to be a prognostic marker of malignancy in several organs, including the oral cavity^14^, there is limited evidence of the success of DNA aneuploidy cytology using brushings as an adjunctive tool for the noninvasive detection of oral cancer^15,16^. In particular, the current evidence must be interpreted prudently for the following reasons: small sample size, heterogeneity of the enrolled subjects, and the different classification criteria used for DNA aneuploidy^16^. Hence, additional well-designed studies are required to evaluate the diagnostic value of DNA-ICM using brushings for OPMD and early OSCC.

We have previously reported that DNA-ICM aided in the diagnoses of high grade dysplasia and oral leukoplakia staging in a small series of cases^17,18^. Regarding the aforementioned limitations, the aim of this study was to elucidate whether DNA aneuploidy was an independent discriminator for carcinoma in OPMD patients, when combined with clinical features by logistic regression in a large prospective series. We also aimed to further establish and validate a risk model based on DNA aneuploidy for the detection of oral cancer.

## Materials and methods

### Patients and the cytobrush procedure

This study was approved by the Institutional Review Board of Shanghai Ninth People’s Hospital [Approval No. SH9H-(2012)21] and written informed consent was obtained from all participating patients. This study was also registered in the Chinese Clinical Trial Registry (ChiCTR-DDD-17013359). In this study, patients exhibiting clinical aspects of the OPMD lesions (i.e., oral leukoplakia, erythroplakia, submucous fibrosis, lichen planus, and lichenoid lesions) who visited the clinic at the Department of Oral Mucosal Diseases, Shanghai Ninth People’s Hospital, Shanghai Jiao Tong University School of Medicine, were prospectively enrolled from March 2013 to December 2018.

The patients were divided into 2 independent sets in a 3:1 ratio^19^: 1) 608 patients enrolled between March 2013 to August 2017 constituted the training set; and 2) 202 patients enrolled between September 2017 and December 2018 constituted the test set. The clinical aspects of the OPMD lesions were classified as either homogenous or non-homogenous types. Flat, thin, uniform, reticular, papular, and plaque patterns were classified as the homogenous type. Verrucous, speckled, nodular, and atrophic and erosive patterns were classified as the non-homogenous type. Moreover, the lesion site was divided into a lateral/ventral tongue and others, and the patients’ age was categorized as either > 60 or ≤ 60-years-old, based on the results of our previous study^20^.

Before scalpel biopsy of the lesion was performed, each patient underwent a cytobrush biopsy at the same location of the lesion. The brush sample was collected by performing brushing of the whole lesion with a liquid-based brush kit (MotiSavant, Motic, Xiamen, China). Next, the scalpel biopsy was then taken from the same location as the brushing. The biopsies were fixed in formalin, embedded in paraffin, and processed for routine histopathological examination at the Department of Oral Pathology at our hospital. Histological diagnoses were performed by 2 oral pathologists blinded to the DNA content results, in accordance with the definition and classification system previously described^7,21^. The inclusion and exclusion criteria for the patients were as follows: inclusion criteria, primary diagnosis of OPMD or OPMD concomitant suspicious OSCC; and exclusion criteria, i) primary diagnosis of OSCC with no history of OPMD, and ii) patients with a history of malignancy.

### DNA-ICM analysis

The DNA-ICM device and cytobrush kit are shown in **Supplementary Figure S1**. The DNA content status was analyzed using ICM as previously described^17,18^ and in accordance with the manufacturer’s protocol (MotiSavant). DNA-ICM analyses were conducted by individuals (C.L. and Y.Z.) who were blinded to the histopathological results, because the DNA content analyses were completed before the histopathological diagnoses. The inconsistent criteria of DNA aneuploidy using brushings in the diagnosis of OSCC have recently been summarized^16^. In the majority of previous studies, an outside DNA index (DI) of 1.8–2.2 and 3.6–4.4 and/or 9c events was defined as aneuploidy, whereas more than 4 or 5 cells with a DI > 2.3 was defined as DNA aneuploidy in some other studies^16^. Consequently, in the current study, we addressed the optimal cut-off value of DNA aneuploidy to establish a risk model for detecting carcinoma within OPMD in a large prospective study.

### Statistical analysis

This diagnostic study was reported as per the STARD checklist for reporting studies of diagnostic accuracy^22^. The receiver operating characteristic (ROC) curve, area under the curve (AUC), and associated 95% confidence interval (CI) were conducted to evaluate the diagnostic value of DNA aneuploidy. The Youden index, defined as the overall correct classification rate minus 1 at the optimal cut-off point, was used as another important index. The optimal cut-off thresholds were determined using the maximum Youden index^23,24^. Logistic regression was used to evaluate the odds ratio (OR) and association among the variables. The risk score of each significant variable was determined according to the β coefficient^25^. Statistics, including the sensitivity and specificity with 95% CI were calculated to determine the diagnostic accuracy of aneuploidy. Statistical analysis was performed using SPSS statistical software for Windows, version 21.0 (SPSS, Chicago, IL, USA). All tests were 2-sided, and *P* values of < 0.05 were considered to be statistically significant.

## Results

### Characteristics of the enrolled patients

**Figure 1:**
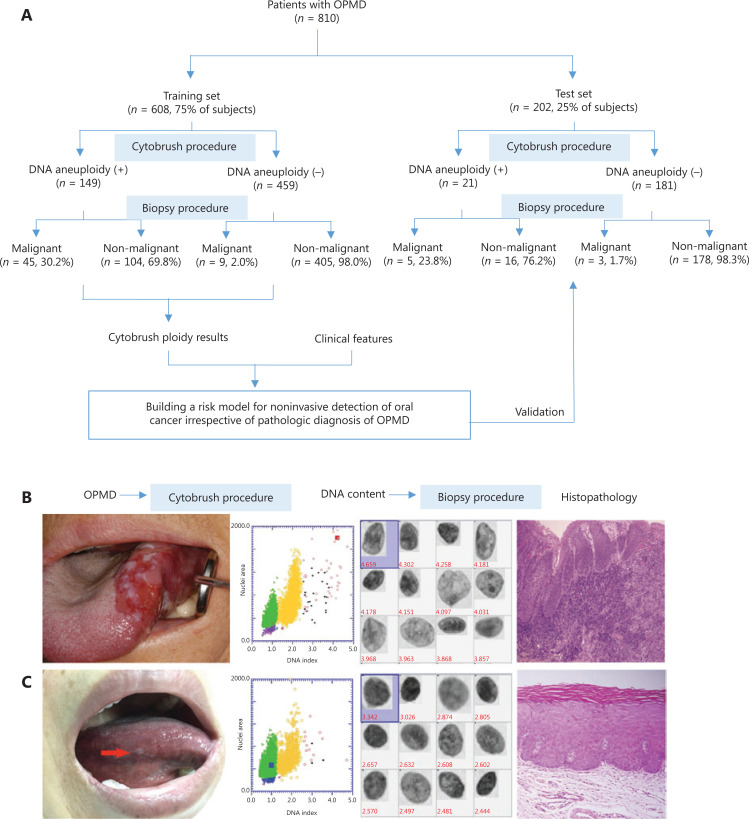
Overview of the workflow of oral potentially malignant disorder (OPMD) patients. (A) The sketch map of the current study. (B) Representative clinical manifestation. The outside DNA index (DI) values determined by DNA-image cytometry, and histopathology of 2 cases of OPMD. A case of a non-homogenous OPMD lesion with DI ≥ 3.5 was determined to be an oral carcinoma. (C) A case of a homogenous OPMD lesion with DI < 3.5 was determined to be an oral dysplasia.

In this prospective diagnostic study, a total of 810 consecutive patients with OPMD were enrolled and divided into 2 independent sets. The flowchart of this study is shown in **Figure 1A**. The baseline characteristics of the training set are listed in **Table 1**. Representative clinical manifestation, DI values determined by DNA-ICM, and histopathology of 2 representative cases of OPMD are shown in **Figure 1 and Supplementary Figure S2**.

**Table 1:** Risk assessment of clinical features and DNA aneuploidy for 608 oral potentially malignant disorder patients recruited from March 2013 to August 2017 in the training set

Characteristic	General OPMD	OPMD concomitant OSCC	Univariate analysis	Multivariate analysis	β coefficient
Total, *n* (%)	554 (91.1)	54 (8.9)	OR (95% CI)	OR (95% CI)	
Age group (years)			*P* = 0.055		
≤ 60	407 (92.5)	33 (7.5)	1.0 (ref)		
> 60	147 (87.5)	21 (12.5)	1.76 (0.99–3.14)		
Gender			*P* = 0.662		
Female	270 (90.6)	28 (9.4)	1.0 (ref)		
Male	284 (91.6)	26 (8.4)	0.88 (0.51–1.54)		
Smoking			*P* = 0.020	*P* = 0.055	
Never	339 (93.4)	24 (6.6)	1.0 (ref)	1.0 (ref)	
Past/present	200 (87.7)	28 (12.3)	1.98 (1.12–3.51)	2.91 (0.98–8.66)	
Alcohol drinking			*P* = 0.005	*P* = 0.499	
Never	346 (93.8)	23 (6.2)	1.0 (ref)	1.0 (ref)	
Past/present	193 (86.9)	29 (13.1)	1.31 (0.74–2.33)	1.44 (0.50–4.14)	
Lesion site			*P* < 0.001	*P* < 0.001	
Others	419 (97.7)	10 (2.3)	1.0 (ref)	1.0 (ref)	1.0 (ref)
Lateral/ventral tongue	135 (75.4)	44 (24.6)	13.66 (6.69–27.87)	7.18 (3.02–17.06)	Risk score = 1.97
Lesion type			*P* < 0.001	*P* < 0.001	
Homogenous	425 (97.0)	13 (3.0)	1.0 (ref)	1.0 (ref)	1.0 (ref)
Non-homogenous	129 (75.9)	41 (24.1)	10.39 (5.40–19.99)	7.67 (3.48–16.88)	Risk score = 2.03
DNA aueuploidy			*P* < 0.001	*P* < 0.001	
DI < 3.5	450 (98.0)	9 (2.0)	1.0 (ref)	1.0 (ref)	1.0 (ref)
DI ≥ 3.5	104 (69.8)	45 (30.2)	21.64 (10.25–46.53)	13.04 (5.46–31.14)	Risk score = 2.57

CI, confidence interval; DI, DNA index; OPMD, oral potentially malignant disorder; OSCC, oral squamous cell carcinoma; OR, odds ratio.

### Optimization of the aneuploid DI value

**Figure 2:**
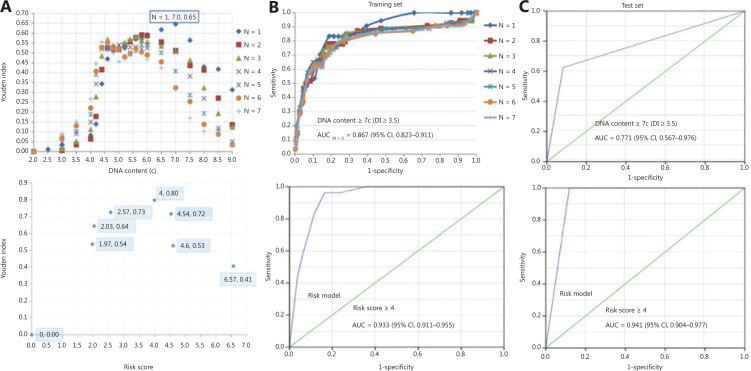
Diagnostic efficacy of DNA aneuploidy and a risk model for oral potentially malignant disorders. (A) The optimal cut-off values of DNA content and risk score by using the maximum Youden index in aneuploidy and risk models, respectively. (B) In the training set, a receiver operating characteristic (ROC) curve of different numbers of DNA content values and risk models, respectively. (C) In the test set, the ROC curve of the DNA content and risk model, respectively. The “N” values represented by the colored symbols denote the numbers of aneuploid cells.

In diagnostic studies, the AUC serves as an overall measure of the accuracy of a particular diagnostic test. The optimal criterion for cut-off point selection in the context of a ROC curve analysis is the maximum of the Youden index^23,24^. To address the optimal cut-off DI values of aneuploidy cytology in detecting carcinoma in OPMD patients, a ROC curve with an AUC analysis was performed using the maximum Youden index for the training set (**Figure 2A**). For the optimal cut-off of at least 1 aneuploid cell with a DI ≥ 3.5 (DNA content ≥ 7.0c), the AUC was a maximum of 0.87 (95% CI: 0.82–0.91; **Figure 2B**). The pair of sensitivity and specificity proportions that corresponded to the Youden index-based cut-off point characterized the performance of the diagnostic test^24^. Thus, the sensitivity and specificity of detecting carcinoma in OPMD patients were 83.3% and 81.2%, respectively.

### Logistic regression and risk score analysis

To clarify the potential confounding variables in the training set, an OR analysis by logistic regression was used to assess the association between DNA aneuploidy and various clinical factors for detecting carcinoma in OPMD patients (**Table 1**). The logistic analysis revealed that age group, gender, smoking, and alcohol consumption did not reach statistical significance, despite being potential confounding variables. Multivariate analyses revealed that lateral/ventral tongue and the non-homogenous type of OPMD were significantly associated with malignant risk (*P* < 0.001), and DNA aneuploidy with a DI ≥ 3.5 in OPMD patients was strongly associated with malignant risk (adjusted OR: 13.04; 95% CI: 5.46–31.14). According to the β coefficient^25^, the risk score for lateral/ventral tongue, non-homogenous type, and DI ≥ 3.5 were 1.97, 2.03, and 2.57, respectively (**Table 1**). The corresponding reference features, other sites, homogenous type, and DI < 3.5 had a risk score of 0.

### Construction of the risk model in the training set

According to the 3 significant independent variables (lateral/ventral tongue, non-homogenous type, and aneuploidy) using multivariate logistic regression, the total score of each patient in the training set represented the sum of scores of the 3 variables, which ranged from 0–6.57. To address the optimal cut-off score of the risk model in detecting carcinoma in OPMD patients, an ROC curve with an AUC analysis was performed using the maximum Youden index in the training set (**Figure 2A**). When the optimal cut-off of the risk score was ≥ 4, the AUC was a maximum of 0.93 (95% CI: 0.91–0.96; **Figure 2B**), and the sensitivity and specificity of detecting carcinoma within OPMD were improved to 96.3% and 83.6%, respectively (**Table 2**).

**Table 2:** Diagnostic assessment of DNA aneuploidy and modified risk model for 608 OPMD patients recruited from March 2013 to August 2017 constituted the training set

Group	General OPMD (*n* = 554)	OPMD concomitant OSCC (*n* = 54)	Diagnostic accuracy (95% confidence interval)
DI ≥ 3.5	False positive (*n* = 104)	True positive (*n* = 45)	Sensitivity = 83.3% (71.0%–91.2%)
DI < 3.5	True negative (*n* = 450)	False negative (*n* = 9)	Specificity = 81.2% (77.8%–84.3%)
Risk model (score ≥ 4)	False positive (*n* = 91)	True positive (*n* = 52)	Sensitivity = 96.3% (86.7%–99.7%)
Risk model (score < 4)	True negative (*n* = 463)	False negative (*n* = 2)	Specificity = 83.6% (80.2%–86.4%)

DI, DNA index; OPMD, oral potentially malignant disorder; OSCC, oral squamous cell carcinoma.

### Validation of the risk model in the test set

We first assessed the diagnostic accuracy of DNA aneuploidy for detecting carcinoma in OPMD patients in the test set. Consistent with the training set, the AUC was a maximum of 0.77 (95% CI: 0.57–0.97; **Figure 2C**) when the optimal cut-off for at least 1 aneuploid cell with a DI ≥ 3.5 (DNA content ≥ 7.0c), and the sensitivity and specificity of detecting carcinoma within OPMD were 62.5% and 91.8%, respectively. We next validated the risk model in the test set. The AUC was a maximum of 0.94 (95% CI: 0.90–0.98) when the optimal cut-off of the risk score was ≥ 4 (**Figure 2C**), and the sensitivity and specificity of detecting carcinoma within OPMD were improved to 100% and 88.1%, respectively (**Table 3**).

**Table 3:** Diagnostic assessment of DNA aneuploidy and modified risk model for 202 patients recruited from September 2017 to December 2018 constituted the test set

Group	General OPMD (*n* = 194)	OPMD concomitant OSCC (*n* = 8)	Diagnostic accuracy (95% confidence interval)
DI ≥ 3.5	False positive (*n* = 16)	True positive (*n* = 5)	Sensitivity = 62.5% (30.4%–86.5%)
DI < 3.5	True negative (*n* = 178)	False negative (*n* = 3)	Specificity = 91.8% (86.9%–94.9%)
Risk model (score ≥ 4)	False positive (*n* = 23)	True positive (*n* = 8)	Sensitivity = 100%
Risk model (score < 4)	True negative (*n* = 171)	False negative (*n* = 0)	Specificity = 88.1% (82.9%–92.0%)

DI, DNA index; OPMD, oral potentially malignant disorder; OSCC, oral squamous cell carcinoma.

## Discussion

DNA aneuploidy is an indicator of numerical chromosomal changes, and its emergence is typically an early crucial step in carcinogenesis^26^. In addition, the hypothesis that DNA aneuploidy serves as a marker of oral cancer progression is of scientific significance^27,28^. Indeed, DNA aneuploidy measured in formalin-fixed paraffin-embedded biopsies appears to have a predictive capacity for the malignant transformation of OPMD^14^. Although DNA aneuploidy cytology using oral brushings may represent a potential noninvasive adjunctive diagnostic tool in the early detection of oral cancer, current evidence is limited mainly by small sample size, heterogeneity of the enrolled patients, and the different classification criteria of aneuploidy used in previous studies^15,16^. To the best of our knowledge, the sample size (*n* = 810) of the current study was the largest-scale series in a single study investigating the diagnostic value of the DNA-ICM using brushings for oral cancer detection within the homogeneity of enrolled patients with OPMD.

Previous studies have reported a wide range of sensitivities (16.0%–96.4%) and specificities (66.6%–100%) of DNA-ICM in screening OPMD using brushings (reviewed in ref. 15), which is thought to be due to variations in study design and aneuploidy criteria^15,16^. First, differences in the criteria used for the inclusion and exclusion of study patients may produce different results. Moreover, the sample sizes in the majority of previous studies were small^16^. Notably, various sample sizes of OSCC, OPMD, and benign lesions enrolled in a study can produce different results, because the detection of OSCC or OPMD is the outcome. Conceivably, a higher proportion of OSCC and benign lesions, as well as a lower proportion of OPMD would increase the diagnostic sensitivity and specificity for screening OPMD^16^. Although the number of malignant to non-malignant OPMD cases can be superficially imbalanced, the proportion of carcinomas within OPMD patients, termed OPMD concomitant OSCC, was found to be low^29^. Arguably, the proportion (7.7%) of OPMD patients with concomitant OSCC/focal cancer in this study was reasonable, when compared to the high proportion (22.0%–50.0%) of OSCC observed in previous studies^16^. Moreover, this proportion (7.7%) was similar to the proportion (7.9%) of malignant transformation of OPMD identified by meta-analysis^30^.

The diagnostic efficacy (AUC = 0.87) of DNA aneuploidy for the detection of carcinoma within OPMD was determined. The efficacy increased (AUC = 0.93) in the risk model when combined with a significant lesion site and features in the training set. Consistently, the efficacy (AUC = 0.77) of DNA aneuploidy in the test set was also increased (AUC = 0.94) in the risk model. Construction of the risk model based on a cytobrush with a DNA-ICM automatic analyzer for the noninvasive detection of oral cancer was achieved, irrespective of the pathological diagnoses and clinical OPMD subtypes. The findings of the current study revealed that lateral/ventral tongue and non-homogenous type were independent significant indicators for cancer detection in OPMD patients. Consistent with this observation, our previous study on oral leukoplakia revealed that lateral/ventral tongue and non-homogenous type were independent significant indicators of malignant transformation^20^. Moreover, DNA aneuploidy using image cytometry represented an early event and may serve as an independent marker strongly associated with OSCC, in agreement with the aneuploidy results analyzed by flow cytometry^31^. These findings suggest that DNA aneuploidy using brushings could be used as an early indicator of disease before the appearance of clinical signs and symptoms in OSCC patients. Although some molecular biomarkers have been reported to have prognostic value related to oral cancer progression^9,10^, DNA-ICM may serve as a useful noninvasive adjunctive tool for oral cancer screening and as a surveillance mechanism for OPMD progression in cancer.

The original aim of the current study was to first evaluate the diagnostic accuracy of DNA-ICM for the noninvasive detection of carcinoma in OPMD patients in the specific setting of an oral medicine specialist practitioner. Subsequently, we aim to evaluate the practicality of this procedure based on DNA aneuploidy and clinical features (lesion sites and non-homogenous appearance) in general dental and community screenings in future studies, irrespective of the specific clinical and pathological diagnoses of OPMD by oral medicine specialists and pathologists. It is a difficult task to differentially diagnose specific OPMD types, similar to the differential diagnosis of leukoplakia and lichen planus for general dentists and practitioners. Ideally, we hope that general dentists and practitioners can utilize noninvasive tools like DNA-ICM, along with the well-recognized clinical indicators (e.g., lateral/ventral tongue and non-homogenous lesions) for the detection of oral malignant changes in the context of general dental and community screening.

The limitations of this study included the design as a cross-sectional diagnostic study, and that ORs did not represent optimal metrics for studying diagnostic accuracy. Thus, further longitudinal studies with adequate follow-up and clinical endpoints should be conducted to evaluate the efficacy of this risk model as a predictive strategy for the malignant transformation of OPMD. Notably, a deep learning algorithm for a computer-aided oral cancer detection system has been developed to provide an automatic medical image classifier without expert knowledge^32^. Research combining DNA-ICM with other noninvasive techniques (e.g., cytology, microRNA, autofluorescence imaging, and toluidine blue staining^29,33–35^) to improve the test results are also warranted. The clinician may make a decision regarding treatment options based on a panel of diagnostic procedures that could be of prognostic value.

## Conclusions

The results of this large-scale diagnostic study using logistic regression showed that DNA aneuploidy in OPMD patients was an independent marker strongly associated with OSCC. Our established risk model was achieved irrespective of pathological diagnoses and clinical OPMD subtypes. This represented a noninvasive adjunctive tool that combined DNA-ICM (DI ≥ 3.5), lateral/ventral tongue, and non-homogenous lesions, to achieve a favorable diagnostic efficacy for the detection of carcinoma in OPMD patients. A multicenter validation of this risk model should therefore be conducted to obtain further evidence for clinical applications. Longitudinal studies on DNA-ICM using oral brushing samples collected at different time points during follow-up as a surveillance tool for oral cancer progression are also warranted.
