# Supplementary material for: Development and validation of a risk model for noninvasive detection of cancer in oral potentially malignant disorders using DNA image cytometry
Source: Cancer Biol Med. 2021 Aug 15;18(3):763–71. doi: 10.20892/j.issn.2095-3941.2020.0531 (PMC8330543; doi:10.20892/j.issn.2095-3941.2020.0531)
Supplement: Supplementary file 1 [file cbm-18-763-s001.pdf]

Supplementary materials

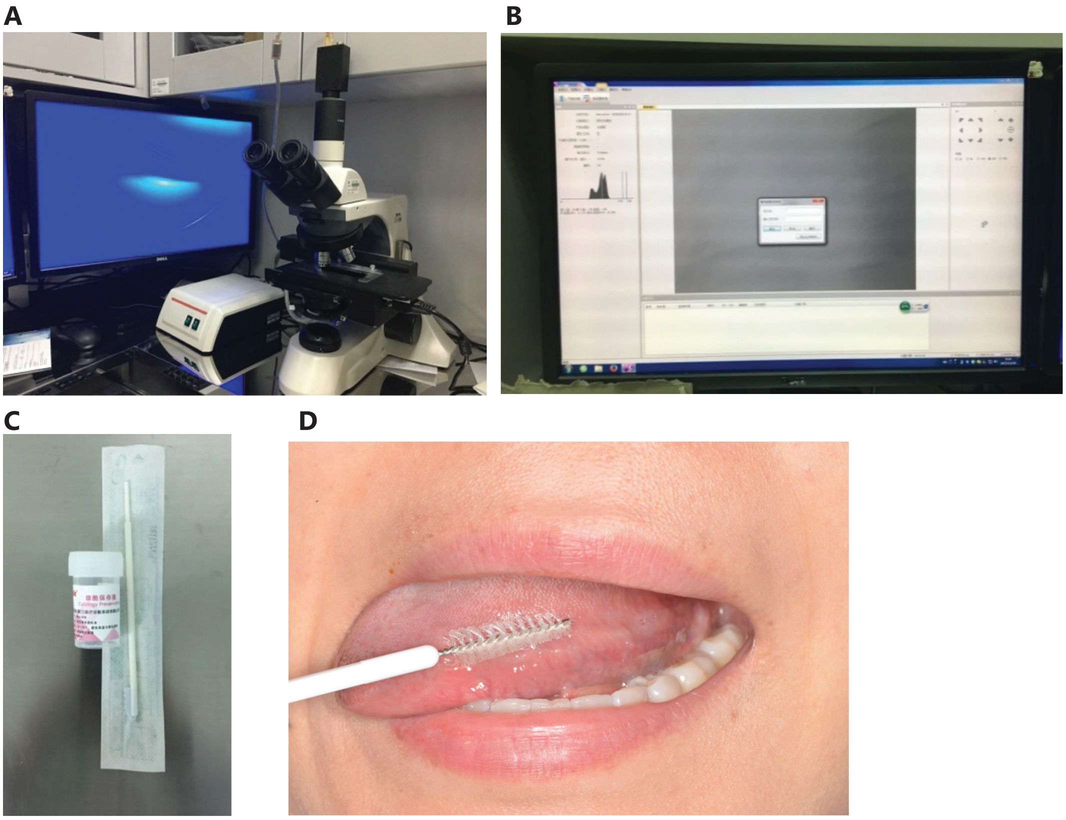

Figure S1 DNA image cytometry (ICM) device and cytobrush kit. (A) The ICM device is an automated DNA image cytometer that connects to the computer. (B) The computer software of imaging analysis (MotiClassify, version 2.1). (C) The liquid-based cytobrush kit. (D) The brush sample was collected by performing brushing of the oral lesion.

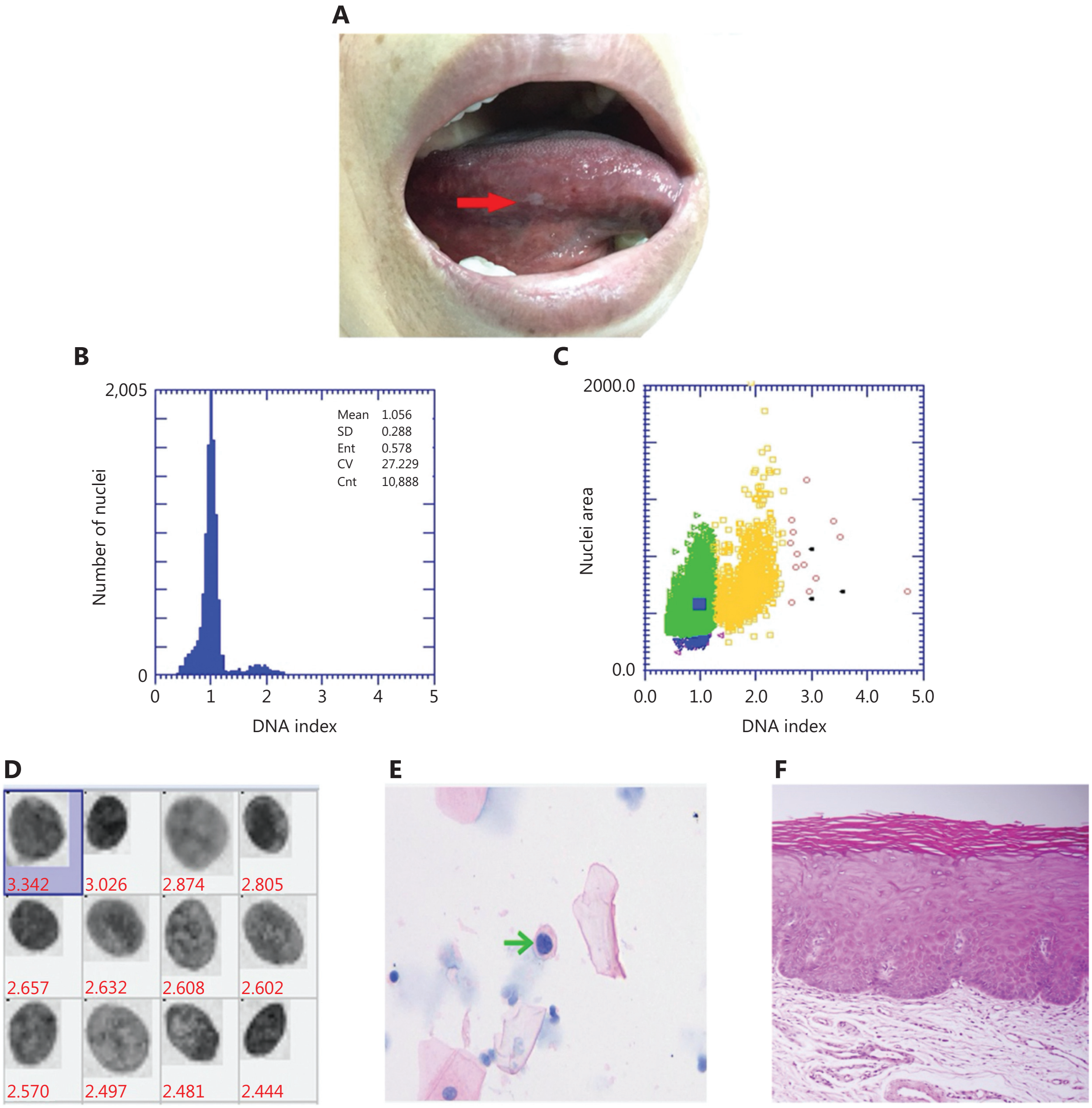

Figure S2 Clinical lesion, DNA content analysis, and histopathological images of a representative case of oral potentially malignant disorder. (A) A white plaque lesion on the right ventral tongue (arrow). (B) A DNA histogram shows 1 G0–G1 peak. (C) A diploid peak (green) is shown in the DNA content scatter plot. The aneuploid cells (empty spots), dysplastic cells (yellow spots), lymphocytes (blue spots), leucocytes (purple spots), and junk cell fraction (black spots) are also presented. (D) A total of 12 cell nuclei (empty spots in C) with DNA index < 3.5. (E) Cytological morphology of a cell nucleus (DNA index = 3.342) in D. (Feulgen-eosin staining, magnification, ×200. Arrow: borderline nuclear changes). (F) Histopathological examination denotes epithelial dysplasia (hematoxylin-eosin staining, magnification, ×100).
